# “Managing the Binder”: A Systematic Review of the Burdens Experienced by Patients and Carers in Managing Personal Health Information Across Fragmented Healthcare Systems

**DOI:** 10.3390/healthcare14182992

**Published:** 2026-09-13

**Authors:** Akshay Soni, Marcel Chua, Warren M. Rozen, Lisa Ellis

**Affiliations:** 1 Department of Plastic and Reconstructive Surgery, Peninsula University Hospital, Frankston, Melbourne, VIC 3199, Australia; 2 Faculty of Medicine and Surgery, Central Clinical School, Monash University, Melbourne, VIC 3004, Australia

**Keywords:** personal health information management, burden of treatment, patient work, caregiver burden, care fragmentation, health information technology, interoperability, patient-held records

## Abstract

**Background**: Fragmented healthcare systems require patients and informal carers to collect, organise, transfer, and interpret personal health information across providers. This review examined the burdens of this work, its management methods, and the evidence for digital tools intended to reduce it. **Methods**: MEDLINE, Embase, CINAHL, and the Cochrane Library were searched for English-language primary studies published from January 2000 to 25 June 2026. Eligible studies examined patient- or carer-led management of personal health information in community, outpatient, or longitudinal care. Two reviewers independently screened records. The findings were synthesised narratively because of study heterogeneity. **Results**: Eleven studies published between 2005 and 2021 were included: seven from the United States, two from Italy, and one from Canada and Israel each. Ten used qualitative methods and one used mixed methods. Patients and carers managed medications, appointments, test results, correspondence, medical histories, and insurance information using folders, binders, handwritten notes, calendars, spreadsheets, memory, and, less commonly, digital portals. Reported burdens included time, administrative effort, cognitive demand, emotional distress, and responsibility for maintaining continuity between services. Burdens were greatest when information demands increased while illness reduced a patient’s capacity to manage them. Paper-based systems remained common because they were visible, portable, and easy to annotate. No study directly tested whether a digital intervention reduced information management burden, and none were conducted in Australia. **Conclusions**: Personal health information management (PHIM) is substantial but largely unrecognised patient and carer work arising partly from fragmented information exchange. The available evidence identifies design considerations for information management, but it does not establish that digital interventions can reduce burden.

## 1. Introduction

The Australian healthcare system delivers care across fragmented public and private networks, encompassing general practitioners (GPs), specialist clinics, hospital services, pathology and imaging providers, and allied health services. Effective communication between these providers is fundamental to safe, coordinated care for people with complex or chronic needs, yet patient health information is routinely dispersed across multiple sites, with no single provider holding a comprehensive record.

My Health Record (MHR), Australia’s national digital health record platform, was introduced in 2012 to address this fragmentation through a centralised, accessible record. Despite almost universal registration from general practices and public hospitals, clinician engagement has remained limited, with only about one in ten medical specialists actively using the system [1]. After the platform moved to an ‘opt-out’ model, a national survey of 2240 Australians reported that 71.2% had limited knowledge of MHR and 29.4% had opted out, with greater awareness associated with higher educational status and chronic disease [2]. Usability challenges have also been identified for individuals with lower e-health literacy [3], and the Australian Digital Health Agency’s 2024 Impact Analysis reported that approximately 20% of consumer complaints between 2019 and 2024 related to pathology and imaging results not appearing within MHR [4].

The cumulative burden of this fragmentation falls disproportionately on patients and their carers, who are increasingly required to manage, coordinate, store, and retrieve their own records to bridge communication gaps between providers. This workload is a predictable result of how the system is structured, and it is particularly demanding for people with complex or chronic conditions who require input from multiple providers. Patients with chronic conditions are the primary managers of their own comorbidities, spending approximately 5800 waking hours per year managing their health [5]. Existing self-management programmes, such as the Chronic Disease Self-Management Program, have shown that patients can be supported to manage their conditions more effectively [6]; however, these have focused on health behaviours and self-efficacy and do not account for the administrative workload imposed by the fragmented systems.

The existing literature has described this as the “invisible work” of personal health information management, in which patients and carers become responsible for managing appointments, transferring results, reconstructing medical histories, maintaining medication lists, and ensuring continuity of information between encounters [7]. Patients often rely on ad hoc systems, such as folders, binders, handwritten notes, screenshots, or memory to manage these burdens.

Two theoretical frameworks underpin this study. The Burden of Treatment Theory (BoTT) proposes that as more care moves outside the clinic, patients and carers take on increasing work, including understanding diagnoses, managing medications, coordinating appointments, and maintaining continuity of information, which forms part of the “burden of treatment” that may eventually exceed their ability to manage it [8]. The Cumulative Complexity Model (CuCoM) expands this by describing the balance between a patient’s workload and their capacity, where accumulating clinical, administrative, and social demands compete against a patient’s physical, cognitive, emotional, and social resources; when workload exceeds capacity, this complexity increases, and outcomes may worsen as a result [9]. Within both frameworks, PHIM is a significant and potentially modifiable source of burden rather than a minor administrative task.

In the Australian context, and despite international recognition of PHIM as a significant form of patient and caregiver workload [10], limited qualitative research has examined how patients and carers navigate fragmented systems in routine encounters, which is increasingly relevant as patients commonly move between public and private providers. In 2015, the OECD described the Australian health system as “too complex for patients”, identifying fragmentation and poor care coordination as the primary reasons [11], with approximately 50% of Australians living with at least one chronic condition, making multi-provider care routine [12]. Australian qualitative studies have found patients with chronic conditions struggling to access care, needing to actively navigate across providers, and describing the absence of a coherent record as a source of frustration and redundancy, with coordination responsibility frequently falling on patients and family members [13,14].

This systematic review therefore aimed to synthesise the available evidence on: (1) PHIM activities undertaken by patients and informal carers; (2) the administrative, cognitive, emotional, and practical burdens associated with this work; (3) the methods and tools used to organise, store, and transfer information; and (4) whether digital tools have been formally evaluated in relation to these burdens, as well as the design requirements for such tools reported by patients and carers. A secondary aim was to assess the relevance of the existing evidence to the Australian context and identify priorities for future research.

## 2. Methods

This is a systematic review of the published literature on PHIM burden in patients with chronic or complex conditions and their informal carers, and of digital health interventions aimed at reducing it. It was designed and reported in accordance with the Preferred Reporting Items for Systematic Reviews and Meta-Analyses (PRISMA 2020) guidelines. The protocol was registered on PROSPERO before the database search (registration number: CRD420261435573).

### 2.1. Eligibility Criteria

Peer-reviewed primary studies reporting original data and published in English from January 2000 to 25 June 2026, the period chosen to capture the literature that has risen during the expansion of electronic health records, patient portals, and other consumer health technologies. Eligible populations included patients, health consumers, informal carers, or family members involved in PHIM, including people with chronic, complex, multimorbid, oncological, surgical, paediatric, or geriatric care needs. Studies were included if they addressed the nature, extent, or consequences of patient- or carer-led PHIM, including record keeping, storage, retrieval or transfer, care coordination, or other related administrative work.

Studies were excluded if they focused exclusively on administrative work performed by clinicians or healthcare organisations without a patient or carer component. Studies restricted to inpatient care were excluded unless they examined ongoing outpatient, community-based or longitudinal information management. Opinion articles, letters without original data, the grey literature, non-English publications, and digital technologies intended exclusively for clinician use were also excluded.

A systematic search was conducted in MEDLINE via PubMed, Embase, CINAHL, and the Cochrane Library. Search concepts covered patients and carers, personal health information, health record management, information burden, administrative workload, care coordination, patient-held records, personal health records, patient portals, and digital health technologies, adapted to the controlled vocabulary and syntax of each database. Terms were combined using Boolean operators, a reproducible strategy corresponding to these search concepts is provided in Supplementary Table S1.

### 2.2. Study Selection

**Figure 1:**
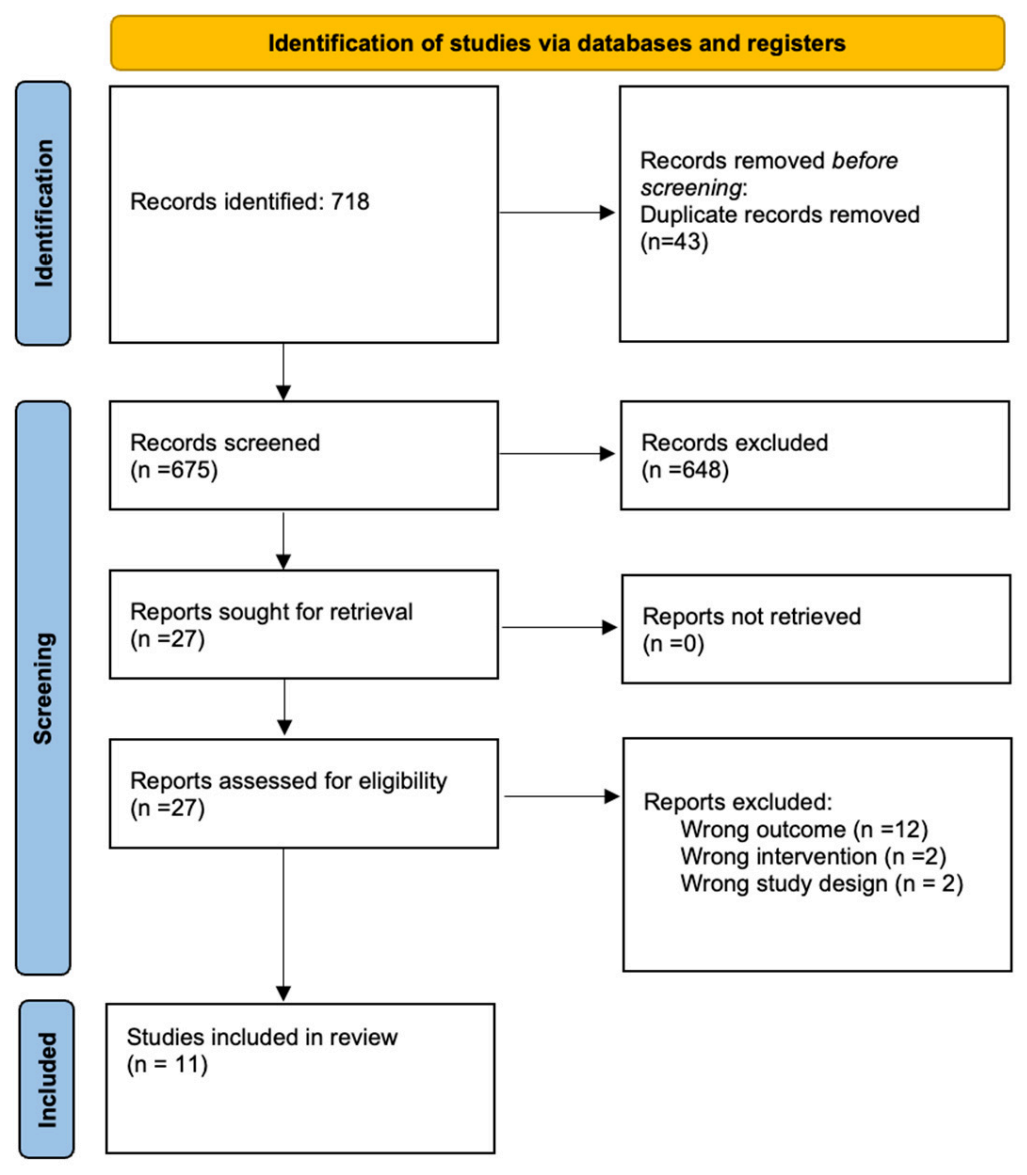
PRISMA 2020 flow diagram for included studies.

All records were imported into the Covidence systematic review software and any duplicates were removed. Two reviewers (A.S. and M.C.) independently screened titles and abstracts against the predefined eligibility criteria, and full-text articles were retrieved for records considered potentially eligible by either reviewer and were independently assessed by both. Disagreements were resolved through discussion and consensus, with a third reviewer available to adjudicate. The reasons for exclusion at the full-text stage were recorded, and the process was documented using a PRISMA flow diagram (Figure 1).

### 2.3. Data Extraction

Data were extracted using a standardised Microsoft Excel form developed for this review. Extracted items included study characteristics (author, year, country, design); population (condition type, carer relationship, setting); the exposure or problem described, including the information management activities studied and how burden was defined; and outcomes, including burden measures used, direction and magnitude of effects, and follow-up durations.

### 2.4. Data Synthesis

Given the heterogeneity of study designs, populations, and outcome measures, a narrative synthesis was conducted, structured around three domains corresponding to the review’s objectives: the characterisation of health information management burden; the current methods of health data management and the work associated with them; and the representation of the Australian healthcare context in the existing literature. The synthesis was guided by either the demands placed on the patients and carers (workload) or the resources available to meet these demands (capacity), following BoTT and CuCoM. The workload–capacity balance was compared across populations of differing clinical complexity in the included studies.

### 2.5. Quality Assessment

Methodological quality was assessed using the Critical Appraisal Skills Programme (CASP) Qualitative Studies Checklist. For mixed-methods studies, CASP was applied to the qualitative component relevant to this review. A summary of appraisal is provided in Table 1 and completed CASP assessment is provided in Supplementary Table S2.

## 3. Results

Eleven studies met the inclusion criteria. They were published between 2005 and 2021 and conducted in the United States (*n* = 7) and Italy (*n* = 2), as well as one in Canada and Israel each. Ten used qualitative approaches, most commonly semi-structured interviews supplemented by home observations, photography of household artefacts, or photo-diaries; one of these employed a nominal group technique with participatory design, and another used a mixed-methods field study. Sample sizes ranged from four families to 88 participants, and the study characteristics are summarised in Table 2. Although the search was conducted up to 25 June 2026, no study was recognised as meeting the eligibility criteria until after 2021. The CASP appraisal showed that all 11 studies stated clear research aims, used appropriate qualitative methodologies and research designs, and addressed questions of value to the field (Table 1). Weakness was observed in five domains. Recruitment was judged to be appropriate in six studies, inappropriate in two, and unclear in three. Researcher–participant relationships or reflexivity were adequately considered in only two of the 11 studies, and ethical issues were adequately reported in three out of 11. Sample sizes were small throughout (four families to 88 participants). However, while similar descriptive themes across studies support the presence and character of PHIM work, these are not sufficient to estimate causal effect.

Findings were synthesised into six PHIM domains: information storage and organisation, information transfer and coordination, memory and cognitive work, carer and household work, emotional burden, and adaptive coping strategies (Table 3). Two studies focused on patients receiving active cancer treatment, while others included people with multiple chronic conditions, cardiac disease, older adults, families managing health information at home, and generally healthy community participants, mostly in outpatient, community or domestic settings. Definitions of PHIM varied, from organising and transferring medical documents to broader activities, such as seeking information, tracking symptoms, managing appointments, and coordinating care.

The information managed was extensive and heterogeneous, typically including medication lists, appointment details, laboratory and imaging results, specialist letters and discharge summaries, medical histories, provider contacts, and insurance or billing information; in the cancer-care studies, this extended to symptoms, side effects, treatment schedules, and questions for clinicians. The tools used were overwhelmingly informal and improvised: memory, handwritten lists, folders, binders, envelopes, plastic bags, calendars, notebooks, spreadsheets, pill bottles, and photographs, alongside less commonly used computers, mobile devices, and patient portals. Moen and Brennan [19] described four differentiated storage strategies organised by anticipated urgency and future use, while Zayas-Cabán [24] documented four families using 22 distinct storage artefacts across nine home locations to complete 69 unique tasks. These findings illustrate the absence of any standardised or system-provided tool and the consequent reliance on ad hoc personal systems (Supplementary Tables S3 and S4).

Patients were the main people undertaking this work, with family members and carers becoming more involved when patients were unwell, older, cognitively impaired, or dependent. Household responsibility was often concentrated in one person; 47 out of 49 self-identified household information managers were women and 31 managed all household health information, while 80% of older participants involved another person in some aspect of this work [19,22].

Burdens were multidimensional, most frequently described in terms of time, administrative effort, cognitive demand, emotional distress, and responsibility for maintaining continuity of care. Participants reported repeated telephone calls, searching for documents, preparing records before appointments, updating lists, following up on missing results, and correcting errors. Ancker et al. [7] characterised the work as imposing time, administrative, cognitive, and responsibility burdens, and emphasised that it was “largely unrecognised.” The mental effort of remembering, reconstructing, and reconciling information was intense when patients were unwell; Klasnja et al. [17] reported that stress, fatigue, pain, nausea, and “chemo brain” impaired concentration and recall precisely when the demands of treatment were greatest. Quantitative estimates of time were rarely reported. Turner et al. [22] provided the clearest quantitative picture, reporting that 44.3% of older adults never tracked health information, 28.4% did so sometimes, and 27.3% did so consistently, as well as that 31% only updated emergency information when something changed while 17% had not updated it since records were created or for more than ten years.

Emotional effects included frustration, anxiety, anger, exhaustion, distress, and feeling overwhelmed; some participants avoided opening or organising information because it was emotionally difficult while others worried that incomplete records might affect their care. Notably, not all patients experienced the work as burdensome. MacGregor and Wathen [18] found that 20 out of 30 participants (67%) had never perceived their health activities as burdensome, and that 18 (60%) preferred “personal health management” to “health work.” This study, however, sampled generally healthy adults with minimal clinical complexity, contrasting the chronic-disease and cancer populations in which burdens are most pronounced.

## 4. Discussion

This systematic review synthesised eleven qualitative and mixed-methods studies of the burdens patients and carers experience in managing personal health information. Across different conditions and settings, patients and families performed substantial, largely unrecognised work to collect, organise, update, transport, and interpret health information. Burdens were greatest among patients with complex illness and the family members supporting them. The main explanation was poor information exchange between providers, as when clinical systems did not communicate, patients became the practical link between services, carrying records between institutions, reconstructing medical histories, and following up on missing results [20,21]. PHIM is therefore not simply individual organisation; it is partly a response to structural fragmentation.

This is consistent with BoTT, in which patients and families absorb increasing healthcare work as care extends beyond the clinic [8], and with the CuCoM, which explains why this becomes burdensome when demands exceed a patient’s available capacity [9]. Through interpretations using the workload–capacity framework, PHIM burden appeared to increase with clinical complexity. Patients with cancer, multimorbidity, or age-related cognitive changes faced greater information demands while their capacity was reduced by fatigue, pain, treatment effects, or memory impairment [7,17,22,23]. In household studies, this workload was often redistributed to family members or carers [19,20,24]. In contrast, generally healthy adults with lower information demands and preserved capacity rarely perceived PHIM as burdensome [18]. These findings suggest that burden arises when information management demands exceed available patient or household resources, rather than being inherent to PHIM itself. Interventions should therefore aim to reduce workload, not merely improve access to information.

Another consistent theme was the invisibility of this work, which occurred outside formal clinical settings and was largely unrecognised by clinicians [7,16,20]. It was ‘opaque’ rather than hidden, incorporated into everyday household routines rather than undertaken as a defined task [20]; recognising it matters, because interventions designed without understanding these practices may add tasks rather than reduce workload. Burdens were also distributed unevenly by gender. Household information management was most often undertaken by wives, mothers, or adult daughters [19,24], suggesting that the workload created by fragmented care may fall disproportionately on women as unpaid and largely invisible labour. This raises both equity and design concerns. Digital tools that assume the availability of a single family member to manage, curate, or share information may reinforce existing caregiving roles and disadvantage people with limited social support. Future evaluations should therefore assess not only whether total workload is reduced, but also who performs it after an intervention is introduced. However, as the included samples were small and non-representative, the magnitude of these differences cannot be estimated from the present evidence.

Patients and carers developed practical strategies to manage this workload. Paper remained central because it was portable, visible, easy to annotate and could be placed where a task occurred, and patients added information not captured in institutional records, such as personal notes, symptom diaries, and question lists [17,19,20,24]. These paper systems should not be read as simple resistance to technology; they fulfilled functions many digital systems reproduce poorly, including visibility, rapid annotation, portability, task prompting, and flexible organisation. Digital systems should therefore support rather than disregard existing practices, since a central repository alone is unlikely to displace paper and may be maintained alongside it, thus adding instead of reducing work (Table 4).

While digital personal health records have been proposed as a potential solution, prior reviews have identified persistent barriers, including usability, workflow integration, privacy, and perceived value [25]. Importantly, none of the included studies evaluated a digital intervention against a comparator, measured burdens before and after implementation, or used a validated burden instrument (Supplementary Table S4). Proposed features such as interoperability, selective sharing, proxy access, reminders, annotation, and integration of patient-generated information [15,17,20,24] should therefore be considered as design hypotheses. More recent studies have described increasing caregiver use of patient portals alongside persistent difficulties with proxy access [26,27], reinforcing the need to evaluate how digital tools complement, rather than replace, existing household information management practices.

Progress in this field is also limited by inconsistent definitions and measurement of PHIM burden. Definitions of burden across the included studies ranged from time, cognitive, emotional, administrative, and coordination demands, but no validated measure was used despite established instruments existing for related constructs, including the Zarit Burden Interview for caregiver burden [28], as well as the Patient Experience with Treatment and Self-management, Treatment Burden Questionnaire, and Multimorbidity Treatment Burden Questionnaire for treatment burden [29,30,31]. Future work would benefit from a common structure derived from BoTT and CuCoM, in which PHIM burden is specified across four workload domains and set against measured capacities. These domains include a time burden, quantified as hours spent locating, preparing, transporting, and following up on information and captured by diary or time-use methods; cognitive burden, assessed through recall, comprehension, and reconciliation demands using established mental workload instruments; emotional burden, assessed through validated distress and anxiety measures; and administrative and coordination burdens, quantified through countable process indicators including contacts initiated, records re-requested, duplicated investigations and errors corrected. Capacity should be characterised separately, including physical and cognitive function, health literacy, digital access and available carer support. Development of a core outcome set through formal consensus with patients and carers would improve comparability across studies and allow future interventions to be evaluated on whether they genuinely reduce burden.

These findings are relevant to the Australian context, where patients frequently move between general practice, specialist care, hospitals, pathology and imaging services, and public and private providers. Like the included countries, Australia delivers care across multiple providers with documented gaps in information exchange, through its mixed public–private system and divided responsibilities across Commonwealth-funded primary care and state-funded hospital services [11]. Australian qualitative research has already described difficulties navigating multiple services and maintaining continuity across fragmented care [13,14]; this review adds detail by identifying the specific work patients and carers perform to compensate. However, no included study was conducted in Australia or evaluated MHR; the relevance and transferability of findings from this review are inferences from structurally comparable systems rather than direct evidence. Australian studies are needed to examine how formal digital records interact with the binders, handwritten summaries, screenshots, photographs, and spreadsheets that patients continue to maintain, and to examine the clinician perspective, including which patient-held information is most useful during consultations and how it can be incorporated into clinical workflows without creating additional work.

A strength of this review is the consistency of findings across diverse populations and settings, and its attention to the domestic and material aspects of information management often overlooked in evaluations of electronic health records. Several limitations should be acknowledged. The methodological limitations varied between studies, and confidence in individual findings should be interpreted accordingly. Studies that clearly reported recruitment, reflexivity, and ethical approval provide greater confidence than those where these areas were poorly described. Small purposive and convenience samples may also have under-represented patients with the lowest capacity or greatest disengagement, as participants willing to discuss and show their record-keeping practices may have been more organised or engaged. Although similar themes were identified across studies, this consistency should not be interpreted as evidence of precision. The evidence base remains small and largely qualitative and cannot determine how common or severe PHIM burden is or establish its causal determinants. Larger quantitative and population-based studies using agreed outcome measures are needed. Most studies were conducted in the United States, which may limit transferability to the Australian setting. Insurance and billing tasks were prominent in several US studies, whereas Australia’s universal Medicare framework and national MHR infrastructure may alter the type of work involved; however, Australian patients still move between public and private providers, so the underlying burden from incomplete interoperability is likely to remain relevant [1,2,3,4,11]. The definitions of work and burden varied, as burden was rarely the primary outcome, and it was not measured using a validated instrument or through time-use or economic analysis. The lack of eligible studies after 2021 is notable given the rapid expansion of patient portals, telehealth, and consumer digital health. Recent portal-use studies [26,27] have suggested that although the field has continued to evolve, this has not necessarily been toward the direct measurement of PHIM burden. Furthermore, the absence of studies after 2021 is unlikely to be an artefact of search sensitivity, and more plausibly reflects that recent digital health research has concentrated on adoption, usability, and effectiveness of specific platforms rather than on patient- and carer-led information work as a burden.

## 5. Conclusions

The available evidence suggests that PHIM is an important but underrecognised form of patient and carer work, particularly when fragmented care increases information demands while illness or dependency reduces capacity. However, the current evidence is insufficient to estimate prevalence or magnitude, establish causal determinants, or show that digital tools can reduce burden. Existing studies have prompted a set of design hypotheses and measurement priorities. Future research should quantify PHIM workload; examine inequities in those who undertake it; and use longitudinal, economic, and co-designed evaluations to determine whether interventions genuinely reduce work instead of redistributing it.

## Figures and Tables

**Table 1:** Summary of CASP quality appraisal.

CASP Domain	Yes	No	Cannot Tell
Clear statement of aims	11	0	0
Qualitative methodology appropriate	11	0	0
Research design appropriate to aims	11	0	0
Recruitment strategy appropriate	6	2	3
Data collected to address research issue	10	1	0
Researcher–participant relationships	2	9	0
Ethical issues adequately considered	3	8	0
Data analysis sufficiently rigorous	11	0	0
Clear statement of findings	8	3	0
Research of value to the field	11	0	0

**Table 2:** Characteristics of included studies.

Author, Year	Country	Design	Sample Size	Study Aim	Clinical Complexity
Ancker et al., 2015 [7]	United States	Qualitative semi-structured interview study using grounded theory analysis	22 patients and 7 healthcare providers	To explore how patients with multiple chronic conditions manage and share personal health information.	Mean of 3.5 (SD 1.5) chronic conditions and regular relationships with an average of 5 providers
Civan et al., 2006 [15]	United States	Qualitative nominal group technique and participatory design study	7 health consumers: 3 in Group 1 and 4 in Group 2	To identify prominent personal health information management activities and consumer preferences for supportive technology.	NR
Bruni et al., 2019 [16]	Italy	Qualitative study using semi-structured interviews and template analysis	44 interviews: patient-only (11), caregiver-only (15), and patient–caregiver interviews (18)	To examine patients’ and relatives’ invisible work in building, mending and coordinating care networks.	Older adults with chronic conditions—15 autonomous; 14 non-autonomous, mainly supported by informal caregivers; and 15 non-autonomous, mainly supported by institutional services
Klasnja et al., 2010 [17]	United States	Six-week qualitative field study using repeated interviews and clinic observations	15 women with breast cancer, aged 37–73 years (mean 50, median 51)	To examine information work performed without adequate informational, physical or attentional resources during cancer treatment.	Active breast cancer treatment; 11 received chemotherapy, 7 surgery, 3 radiotherapy, and 1 hormone therapy
MacGregor and Wathen, 2014 [18]	Canada	Exploratory qualitative semi-structured interview study	30 participants: 14 men and 16 women, aged 21–75 years	To explore perceptions of time, burden, work and responsibility associated with personal health management.	Generally healthy adults; some reported minor, non-life-threatening, or previously serious conditions
Moen and Brennan, 2005 [19]	United States	Descriptive exploratory study using semi-structured interviews and photographs of household artefacts	49 self-identified household information managers	To characterise health information management in the household and identify implications for consumer health technologies.	Healthy volunteers representing households with prevention and health management needs, including cardiovascular, respiratory, nutritional, cancer, and mental health concerns
Piras and Zanutto, 2010 [20]	Italy	Qualitative study using semi-structured interviews, home observations, and photographs	32 families; 42 interviews	To examine household medical-document management to inform personal health record design.	16 families had children aged under 14; 16 had at least one member with a chronic condition requiring a GP and a specialist
Rassin et al., 2007 [21]	Israel	Qualitative study using semi-structured interviews and content analysis	28 patients; aged 30–76 years; 17 were men and the rest were women	To examine how patients perceive, keep, and manage medical documents.	Ischemic heart disease and/or rhythm disorders
Turner et al., 2021 [22]	United States	Mixed-methods field study using interviews, surveys, and a follow-up feedback survey	88 completed interviews; 38 completed the feedback survey	To understand older adults’ personal health information management needs and practices to inform health information technology design.	Generally healthy adults aged 60–98 years; mean Charlson Comorbidity Index 2.0 ± 1.3
Unruh and Pratt, 2008 [23]	United States	Qualitative longitudinal field study using interviews, collection reviews, and photo diaries	35 cancer patients across three study parts	To identify barriers patients face when organising health information during cancer care.	Cancer patients; parts 2 and 3 followed breast cancer patients during 12 weeks of active treatment
Zayas-Cabán, 2012 [24]	United States	Descriptive case study using interviews, household photographs, home layouts, and task observations	Four families: one single-parent, two nuclear-family, and one extended-family household	To describe families’ health information management tasks and how they were performed in the home.	Varied across families; one had a child with special needs, one household was managing hypertension, and two households reported no major health concerns

**Table 3:** Thematic summary table.

PHIM Domain	Reported Findings	Examples of Tools/Strategies	Reported Burden	Studies
Information storage and organisation	Patients stored records across multiple locations and formats	Patients developed flexible systems suited to their daily routines	Organisational effort, information overload, and difficulty locating records	[7,15,19,20,21,22,23,24]
Information transfer and coordination	Patients and carers transported information between providers and followed up on missing records	Carrying paper records, maintaining copies, appointment bundles, and contacting providers	Time, administrative, and responsibility burdens	[7,16,20,21]
Memory and cognitive work	Patients had to remember medications, appointments, questions, and prior clinical information	Memory, lists, diaries, reminders, and mobile phones	Cognitive load, impaired recall, and difficulty processing information when unwell	[7,15,17,22,23]
Carer and household work	Family members increasingly assumed PHIM responsibilities when patient capacity declined	Shared calendars, proxy management, and family scheduling	Coordination burden, unpaid caregiving, and responsibility burden	[7,16,19,22,24]
Emotional burden	Managing records was sometimes associated with frustration, anxiety, or feeling overwhelmed	Avoidance, assistance from family, and simplified personal systems	Distress, frustration, anxiety, and exhaustion	[7,17,21,23,24]
Adaptive coping strategies	Patients developed flexible systems suited to their daily routines	Visible reminders, portable folders, filing/piling systems, and selective storage	Ongoing maintenance effort, sustained vigilance and burden from persistent fragmentation.	[17,19,20,21,22,23,24]

**Table 4:** Thematic synthesis and implications for future PHIM research and design.

Theme	Synthesis Finding	Implication
Workload and capacity	Administrative, cognitive, emotional, and coordination demands became more salient when illness, dependency or complexity reduced available capacity.	Measure both PHIM workload and patient/carer capacity; stratify by clinical complexity and care transitions.
Paper and household systems	Paper remained useful because it was visible, portable, rapidly annotated, and embedded in household routines.	Digital tools should test equivalent affordances such as rapid annotation, persistent task cues, flexible organisation, printable/portable summaries and selective sharing.
Carer and gendered work	Family members often assumed PHIM when patients had reduced capacity; women predominated among household information managers in several studies.	Measure who performs PHIM, unpaid time and delegation; co-design tools that do not assume an always-available household manager.
Digital evidence gap	No included study tested whether a digital intervention reduced PHIM burden.	Treat current digital features as design hypotheses; evaluate effectiveness using burden outcomes rather than adoption alone.
Transferability	No included study was Australian, and most evidence came from small US samples.	Undertake Australian observational, co-design, longitudinal, and economic studies before assuming international findings apply directly.

## Data Availability

No new data were created or analyzed in this study. Data sharing is not applicable to this article.

## Supplementary Materials

The following supporting information can be downloaded at: https://www.mdpi.com/article/10.3390/healthcare14182992/s1, Table S1: Search Strategy; Table S2: Critical Appraisal using the Critical Appraisal Skills Programme (CASP) Qualitative Studies Checklist; Table S3: Personal health information management work, burden and consequences; Table S4: Coping strategies, digital design features and key findings.
