# Supplementary material for: “Managing the Binder”: A Systematic Review of the Burdens Experienced by Patients and Carers in Managing Personal Health Information Across Fragmented Healthcare Systems
Source: Healthcare (Basel). 2026 Sep 13;14(18):2992. doi: 10.3390/healthcare14182992 (PMC13606204; doi:10.3390/healthcare14182992)
Supplement: Supplementary file 1 [file healthcare-14-02992-s001.zip › healthcare-4523377-supplementary.pdf]

Table S1: Search strategy

	Search concept	Search terms
PubMed/MEDLINE	**Personal health information management**	"Health Records, Personal"[Mesh] OR "Medical Records"[Mesh] OR "personal health information management"[tiab] OR "personal health information"[tiab] OR "health information management"[tiab] OR "managing health information"[tiab] OR "managing medical record*"[tiab] OR "managing their records"[tiab] OR "record keeping"[tiab] OR "record-keeping"[tiab] OR "document management"[tiab] OR "patient held record*"[tiab] OR "patient-held record*"[tiab] OR "personal health record*"[tiab] OR "keeping track"[tiab] OR "organizing information"[tiab] OR "organising information"[tiab] OR "information work"[tiab] OR "invisible work"[tiab]
	**Population**	"Chronic Disease"[Mesh] OR "Multiple Chronic Conditions"[Mesh] OR "Multimorbidity"[Mesh] OR "Caregivers"[Mesh] OR multimorbid*[tiab] OR "multiple chronic"[tiab] OR "chronic condition*"[tiab] OR "chronic illness*"[tiab] OR "complex condition*"[tiab] OR "complex needs"[tiab] OR comorbid*[tiab] OR co-morbid*[tiab] OR carer*[tiab] OR caregiver*[tiab] OR "care giver*"[tiab] OR "family caregiver*"[tiab] OR patient*[tiab]
	**Burden, workload and care coordination**	"Cost of Illness"[Mesh] OR "Workload"[Mesh] OR "Caregiver Burden"[Mesh] OR burden[tiab] OR workload[tiab] OR "invisible work"[tiab] OR "self-management"[tiab] OR "self management"[tiab] OR coordinat*[tiab] OR navigat*[tiab] OR fragment*[tiab] OR "time spent"[tiab] OR effort*[tiab] OR labor[tiab] OR labour[tiab] OR experience*[tiab] OR challenge*[tiab]
	**Limits**	("2000"[Date - Publication] : "3000"[Date - Publication]) AND English[lang]
Embase	**Personal health information management**	(exp "personal health record"/ OR exp "medical record"/ OR "personal health information management".ti,ab. OR "personal health information".ti,ab. OR "health information management".ti,ab. OR "managing health information".ti,ab. OR "managing medical record*".ti,ab. OR "managing their records".ti,ab. OR "record keeping".ti,ab. OR "record-keeping".ti,ab. OR "document management".ti,ab. OR "patient held record*".ti,ab. OR "patient-held record*".ti,ab. OR "personal health record*".ti,ab. OR "keeping track".ti,ab. OR "organizing information".ti,ab. OR "organising information".ti,ab. OR "information work".ti,ab. OR "invisible work".ti,ab.)
	**Population**	(exp "chronic disease"/ OR exp "multiple chronic conditions"/ OR exp caregiver/ OR multimorbid*.ti,ab. OR "multiple chronic".ti,ab. OR "chronic condition*".ti,ab. OR "chronic illness*".ti,ab. OR "complex condition*".ti,ab. OR "complex needs".ti,ab. OR comorbid*.ti,ab. OR co-morbid*.ti,ab. OR carer*.ti,ab. OR caregiver*.ti,ab. OR "care giver*".ti,ab. OR "family caregiver*".ti,ab. OR patient*.ti,ab.)

	**Burden, workload and care coordination**	(exp "cost of illness"/ OR exp workload/ OR exp "caregiver burden"/ OR burden.ti,ab. OR workload.ti,ab. OR "invisible work".ti,ab. OR "self-management".ti,ab. OR "self management".ti,ab. OR coordinat*.ti,ab. OR navigat*.ti,ab. OR fragment*.ti,ab. OR "time spent".ti,ab. OR effort*.ti,ab. OR labor.ti,ab. OR labour.ti,ab. OR experience*.ti,ab. OR challenge*.ti,ab.)
	**Limits**	AND [2000-2026]/py AND [english]/lim
**Cochrane**	**Personal health information management**	[mh "Health Records, Personal"] OR [mh "Patient Portals"] OR "personal health record*":ti,ab,kw OR "patient held record*":ti,ab,kw OR "patient-held record*":ti,ab,kw OR "patient portal*":ti,ab,kw OR "shared care record*":ti,ab,kw OR "shared health record*":ti,ab,kw OR "health information management":ti,ab,kw OR "personal health information":ti,ab,kw OR "record keeping":ti,ab,kw OR "managing health information":ti,ab,kw OR "managing medical record*":ti,ab,kw OR "My Health Record":ti,ab,kw
	**Population**	[mh "Chronic Disease"] OR [mh "Multiple Chronic Conditions"] OR [mh Caregivers] OR multimorbid*:ti,ab,kw OR "multiple chronic":ti,ab,kw OR "chronic condition*":ti,ab,kw OR "chronic illness*":ti,ab,kw OR "complex condition*":ti,ab,kw OR "complex care":ti,ab,kw OR comorbid*:ti,ab,kw OR carer*:ti,ab,kw OR caregiver*:ti,ab,kw OR "family caregiver*":ti,ab,kw
	**Burden, workload and care coordination**	[mh "Cost of Illness"] OR [mh "Caregiver Burden"] OR "treatment burden":ti,ab,kw OR "administrative burden":ti,ab,kw OR "patient burden":ti,ab,kw OR "carer burden":ti,ab,kw OR "caregiver burden":ti,ab,kw OR "invisible work":ti,ab,kw OR "time burden":ti,ab,kw OR "self-management":ti,ab,kw OR "care coordination":ti,ab,kw OR "information transfer":ti,ab,kw OR usability:ti,ab,kw OR "patient experience":ti,ab,kw
	**Limits**	2000–present

Table S2: Critical Appraisal using the Critical Appraisal Skills Programme (CASP) Qualitative Studies Checklist

Critical Appraisal using the Critical Appraisal Skills Programme (CASP) Qualitative Studies Checklist											
	Section A: Are the results valid?						Section B: What are the results?			Section C: Will the results help locally?	Appraisal summary
	Was there a clear statement of the aims of the research?	Is a qualitative methodology appropriate?	Was the research design appropriate to address the aims of the research?	Was the recruitment strategy appropriate to the aims of the research?	Was the data collected in a way that addressed the research issue?	Has the relationship between researcher and participants been adequately considered?	Have ethical issues been taken into consideration?	Was the data analysis sufficiently rigorous?	Is there a clear statement of findings?	How valuable is the research?	
Turner et al., 2021	Y	Y	Y	Y	Y	N	N	Y	Y	Y	**Positive/Methodologically sound**<br>The study design is well described and methodology sound. The study contributes by providing insights into the nature of administrative burden.<br><br>**Negative/Relatively Poor methodology**<br>Minimal description of ethical approval.<br><br>**Unknowns**<br>N/A
Bruni et al., 2019	Y	Y	Y	N	Y	N	N	Y	N	Y	**Positive/Methodologically sound**<br>The study contributes by providing insights into the nature of administrative burden.<br><br>**Negative/Relatively Poor methodology**<br>Due to the nature of the study from a social science perspective, its methodology is indirectly relevant.<br>Minimal description of ethical approval.<br><br>**Unknowns**<br>N/A

Ancker et al., 2015	Y	Y	Y	Y	Y	Y	Y	Y	Y	Y	**Positive/Methodologically sound**<br>Comprehensive description of methodology. The study contributes by providing insights into the nature of administrative burden.<br><br>**Negative/Relatively Poor methodology**<br>N/A<br><br>**Unknowns**<br>N/A
MacGregor and Wathen, 2014	Y	Y	Y	Y	Y	N	Y	Y	Y	Y	**Positive/Methodologically sound**<br>The study contributes by providing insights into the nature of administrative burden.<br><br>**Negative/Relatively Poor methodology**<br>No relationship was described between investigator and participant, contributing to risk of bias.<br><br>**Unknowns**<br>N/A
Zayas-Cabán, 2012.	Y	Y	Y	N	Y	N	N	Y	N	Y	**Positive/Methodologically sound**<br>The study contributes by providing insights into the nature of administrative burden.<br><br>**Negative/Relatively Poor methodology**<br>This study was a case series of four families, providing relatively poor evidence with its low power.<br><br>**Unknowns**<br>N/A
Piras and Zanutto, 2010	Y	Y	Y	Y	Y	N	N	Y	Y	Y	**Positive/Methodologically sound**<br>The study contributes by providing insights into the nature of administrative burden.<br><br>**Negative/Relatively Poor methodology**<br>No relationship was described between investigator and participant, contributing to risk of bias.<br>Minimal description of ethical approval.

											**Unknowns**<br>N/A
Klasnja et al., 2010	Y	Y	Y	Y	Y	N	N	Y	Y	Y	**Positive/Methodologically sound**<br>The study contributes by providing insights into the nature of administrative burden.<br><br>**Negative/Relatively Poor methodology**<br>No relationship was described between investigator and participant, contributing to risk of bias.<br>Minimal description of ethical approval.<br><br>**Unknowns**<br>N/A
Unruh and Pratt, 2008	Y	Y	Y	CT	N	N	N	Y	Y	Y	**Positive/Methodologically sound**<br>The study contributes by providing insights into the nature of administrative burden.<br><br>**Negative/Relatively Poor methodology**<br>Recruitment strategy was not described.<br>No relationship was described between investigator and participant, contributing to risk of bias.<br>Minimal description of ethical approval.<br><br>**Unknowns**<br>N/A
Rassin et al., 2007	Y	Y	Y	CT	Y	Y	Y	Y	Y	Y	**Positive/Methodologically sound**<br>The study contributes by providing insights into the nature of administrative burden.<br><br>**Negative/Relatively Poor methodology**<br>Recruitment strategy was not described.<br><br>**Unknowns**
Civan et al., 2006	Y	Y	Y	CT	Y	N	N	Y	N	Y	**Positive/Methodologically sound**<br>The study contributes by providing insights into the nature of administrative burden.<br><br>**Negative/Relatively Poor methodology**<br>Recruitment strategy was not described.

											No relationship was described between investigator and participant, contributing to risk of bias.<br>Minimal description of ethical approval.<br><br>**Unknowns**
Moen and Brennan, 2005	Y	Y	Y	Y	Y	N	N	Y	Y	Y	**Positive/Methodologically sound**<br>The study contributes by providing insights into the nature of administrative burden.<br><br>**Negative/Relatively Poor methodology**<br>No relationship was described between investigator and participant, contributing to risk of bias.<br>Minimal description of ethical approval.<br><br>**Unknowns**<br>N/A
Y = Yes; N = No; CT = Can't Tell; N/A = Not applicable											

Table S3. Personal health information management work, burden and consequences.

Author, year	Person performing the information work	Type of information managed	Tool or method used (eg binders, notebooks, photos, memory etc)	Source of fragmentation or difficulty	Time/frequency of task	Burden dimension	Emotional and cognitive impact	Consequences for care
**Ancker et al., 2015 [7]**	Patients; sometimes family members, particularly wives or adult daughters	Medication lists, medical history, laboratory results, imaging, appointments, contact details, bills and insurance information.	Memory, handwritten lists, folders, spreadsheets, pill bottles, business cards, paper records and patient portals	Poor interoperability, missing or inaccurate records, insurance errors, pharmacy errors and repeated attempts to contact the correct organisation.	Quantitative time NR; tasks were described as lengthy and often required repeated follow-up	Time, administrative, cognitive and responsibility burden	Frustration, exhaustion, anger, distress, sleep disruption and difficulty remembering information when unwell.	Delayed surgery, incomplete information sharing, suboptimal decisions and failure to pursue care because of the effort required.
**Civan et al., 2006 [15]**	Individual health consumers.	Medical records, medications, appointments, symptoms, health history, laboratory results, referrals, bills, insurance information and family history	Memory, sensory observation, paper records, calendars, diaries, lists, spreadsheets, email, mobile devices, pill boxes and electronic logs	Scattered information, reliance on faulty memory and difficulty obtaining copies of medical records	Quantified time NR; activities included routine prospective tracking and retrospective reconstruction after health events	Cognitive burden, routine effort and information fragmentation.	Participants described memory as unreliable and reported frustration obtaining medical records.	Participants described memory as unreliable and reported frustration obtaining medical records.
**Bruni et al., 2019 [16]**	Patients and relatives/informal caregivers, particularly close family members when patients lacked autonomy	Available services, eligibility, diagnoses, patient autonomy, appointments, medications and care requirements.	Formal and informal information gathering, asking others, coordinating appointments, family scheduling and medication preparation.	Poor coordination between health and social services, vague information, rigid institutional rules, service gaps and differing provider availability.	Overall time NR; care-network management required continuous maintenance. One case reported 55 h per week of formal/private assistance, with remaining care	Administrative, coordination, time, responsibility and practical caregiving burden.	Frustration, loneliness and concern about burdening relatives; cognitive impact NR.	Families filled service gaps, maintained care continuity and reduced risks such as medication confusion and falls. Patients without strong social networks risked insufficient

					performed by the wife.			or inaccessible care.
**Klasnja et al., 2010 [17]**	Patients, sometimes assisted by family, friends or appointment companions.	Symptoms, side effects, questions, clinician advice, appointments, medical history, medications, doses, laboratory results and contact details.	Notes, calendars, folders, appointment bundles, “cancer bags,” medication bottles, business cards, email, mobile phones, Blackberries and post-it notes.	Diminished attention, unfamiliarity with cancer care, mobility, inadequate workspaces and limited access to computers, printers or internet.	Quantified time NR; needs arose during appointments, travel, phone calls, treatment and unexpected daily situations.	Cognitive, time, logistical and mobility-related burden.	Stress, anxiety, fatigue, pain, nausea and “chemo brain” impaired concentration, recall and information processing.	Forgotten questions, missing information, misunderstood advice and one treatment-centre decision based on misunderstanding.
**MacGregor and Wathen, 2014 [18]**	Individual participants managing their own health; some also discussed family health activities.	Health documents, symptoms, blood pressure, appointments and health information obtained from professionals, friends, books and the internet.	Paper or electronic records, internet searching, smartphone apps and computer-generated graphs.	NR	Specific time estimates NR. Information management, tracking and seeking were generally perceived as taking very little or no time; one participant made weekly blood-pressure graphs taking “just seconds.”	Time, effort and perceived obligation. Most participants did not consider personal health management burdensome	Most expressed satisfaction or positive attitudes; some reported pressure, guilt or discomfort with describing health activities as “work.”	NR
**Moen and Brennan, 2005 [19]**	A self-identified household information manager; 47/49 (95%) were women and 31/49 (64%) managed all household health information.	Appointments, insurance, provider contacts, prescriptions, medical schedules, history, immunisations, observations,	Primarily paper tools, including calendars, phonebooks, loose sheets, cards, folders, notebooks, printouts and stored medical	Growing quantities of information, information overload, language barriers and difficulty locating or	Quantified task time NR; daily or frequent-use information was kept visible, while less frequently used information was stored in known locations.	Information-management demand, organisational effort and information overload.	A few participants found information confusing and avoided searching for it; some Latino participants reported difficulty understanding	NR

		laboratory results, X-rays and self-care instructions.	records; computers and personal digital assistants were less common.	understanding information.			available information.	
**Piras and Zanutto, 2010 [20]**	Patients, parents and relatives/informal caregivers	Logbooks, laboratory results, specialist reports, radiographs, scans, discharge letters, vaccination records, medications and symptom records.	Annotations, highlighting, internet printouts, personal diaries, folders, drawers, boxes, calendars and monitoring devices.	Healthcare facilities did not directly exchange information; patients had to collect, organise and present records to clinicians.	Quantified time NR. Records could remain in visible household spaces for weeks to years; active records were incorporated into daily routines.	Coordination, organisational and cognitive burden associated with selecting, sorting, updating and sharing records.	Records carried emotional meaning, including pride, shame and attachment; patients also had to learn clinicians' undocumented expectations.	Missing or poorly organised records could require another appointment or waste consultation time; patient-created information sometimes supported diagnosis.
**Rassin et al., 2007 [21]**	Patients; relatives sometimes retrieved or transported documents.	Hospital summaries, ECGs, imaging, laboratory results, medication information, catheterization reports and specialist documentation.	Envelopes, plastic bags, folders, photocopies and documents stored at home, in cars, workplaces or briefcases.	No communication link between databases; information was transferred between institutions through patients. Patients lacked guidance about which documents to bring.	Quantified time NR. One participant reported spending "a lot of time" preparing documents because finding them was difficult.	Time spent locating records, effort carrying documents and responsibility for selecting relevant information.	Missing records caused anger and discontent; organised folders provided confidence, control and empowerment.	Missing records caused delayed consultations, prolonged clinic visits and repeat examinations. Available records improved communication and visit efficiency.
**Turner et al., 2021 [22]**	Older adults, sometimes assisted by family, friends, facility staff or proxies.	Symptoms, medications, blood glucose, weight, appointments, clinic summaries, insurance claims, emergency contacts, allergies	Memory, paper notes, calendars, files, piles, pillboxes, medical devices, phones, computers, patient portals and other people.	Changing health, memory, living arrangements, social support, digital literacy and available technology.	Tracking was never performed by 44.3%, sometimes by 28.4% and consistently by 27.3%. Emergency information was updated only	Organisational, cognitive, digital and responsibility burden; consistent tracking and emergency-plan updating were difficult.	Some avoided sharing to preserve independence or avoid burdening others; memory decline led some to use proxies.	PHIM supported symptom monitoring, provider communication, decision-making and emergency planning; direct clinical outcomes NR

		and POLST forms.			when something changed by 31%; 17% had not updated it since creation or for over 10 years.			
**Unruh and Pratt, 2008 [23]**	Patients; assistance from caregivers or social networks was recommended	Bills, insurance claims, appointment slips, clinical documents and treatment information.	Unorganised stacks, folders, notebooks, post-it notes and personal health-information collections.	Emotional, scalable, temporal and functional barriers.	Quantified task time NR. Information accumulated continuously during treatment; Parts 2 and 3 lasted 12 weeks.	Emotional, cognitive, organisational and time burden.	Patients described feeling overwhelmed, distressed and anxious, sometimes avoiding information because they "just couldn't deal" with it.	Poor organisation impaired information retrieval and caused missed time-sensitive financial tasks and difficulty using information during treatment.
**Zayas-Cabán, 2012 [24]**	Usually the mother and/or wife as primary health information manager; some tasks were shared with other household members.	Appointments, provider contacts, prescriptions, medication lists, immunisation records, medical history, blood-pressure readings and health literature.	File cabinets, calendars, cupboards, refrigerator doors, portfolios, notebooks, computers, telephones and paper notes.	Coordinating multiple professionals, maintaining records, language barriers, limited technology access, changing insurance, travel to appointments and information stored across multiple locations.	Tasks occurred over 1–6 months of study participation. Some were frequent or routine; others were needs-based. One participant checked blood pressure twice daily and attended appointments every 2–3 weeks.	Coordination, organisational, time, technological and language-related burden.	One mother reported that collecting information was sometimes depressing and felt there was "no way to keep up with it."	Family members missed appointments, and one parent changed career arrangements to attend appointments. Records supported provider communication and monitoring.

Table S4. Coping strategies, digital design features and key findings.

Author, year	Coping strategy	Digital intervention features if any	Key findings
**Ancker et al., 2015 [7]**	Carrying records, keeping lists or spreadsheets, repeatedly checking results, contacting insurers and seeking family support.	NA	Patients used paper, electronic tools and memory to manage fragmented information. Correcting and transferring records created substantial, largely unrecognised work
**Civan et al., 2006 [15]**	Participants described memory as unreliable and reported frustration obtaining medical records.	Proposed centralised tool with user control, selective sharing, integrated information, security, backups, flexible input methods and clinician contributions	PHIM involved monitoring health, creating health histories, decision-making, planning and action. Desired tool principles were control, sharing, integration, security and flexibility.
**Bruni et al., 2019 [16]**	Combining public, private and informal care; recruiting relatives, neighbours or paid carers; dividing shifts; and coordinating appointments and unexpected events.	NA	Patients and caregivers performed three forms of invisible work: building care networks, mending service gaps and coordinating formal and informal caregivers.
**Klasnja et al., 2010 [17]**	Mobile information collections, appointment bundles, immediate capture followed by later sorting, assistance from others, repurposed tools and visible reminders.	NA	Unanchored work involved capturing and retrieving information. Four main causes were diminished attention, unfamiliarity, mobility and inadequate environments; patient workarounds frequently broke down.
**MacGregor and Wathen, 2014 [18]**	NR	NA	20/30 (67%) had never perceived health activities as burdensome; 2/30 (7%) currently considered them burdensome, 5/30 (17%) reported past burden, and 3/30 (10%) were unclear. Eighteen participants (60%) preferred “personal health management” over “health work.”
**Moen and Brennan, 2005 [19]**	“Just-in-time,” “just-at-hand,” “just-in-case” and “just-because” storage strategies based on anticipated urgency and future use	NA	Household information management was usually performed by one person, relied heavily on paper, and used four differentiated storage strategies.
**Piras and Zanutto, 2010 [20]**	Minimum-effort, adaptive and shared-care record-management strategies.	Proposed PHR features included annotations, personal diaries, document integration, selective sharing, caregiver coordination and flexible organisation.	Record keeping was invisible articulation work; patients modified documents, organised them spatially within daily life and used three distinct management strategies.
**Rassin et al., 2007 [21]**	Keeping copies, organising documents by date or category, carrying recent records and selecting documents by perceived importance.	NA	Most patients kept medical documents despite believing this was the health system’s responsibility. Managing and routing records increased involvement, control and empowerment.

**Turner et al., 2021 [22]**	Filing, piling or discarding records; using visible reminders, patient portals, family assistance and proxy management.	NA	Five PHIM activities were identified: seeking, tracking, organising, sharing and emergency planning. Most participants (80%) involved others, and approaches varied with health, living situation, social support and technology use.
**Unruh and Pratt, 2008 [23]**	Storing information in folders or piles, creating notebooks and post-it reminders, and seeking help from others.	NA	Four barriers hindered organisation: emotional reactions, increasing information volume, limited time and lack of understanding about future information use.
**Zayas-Cabán, 2012 [24]**	Portfolios, shared calendars, visible reminders, paper logs, family assistance, interpreters and established household routines.	Proposed features included multi-user access, permissions, multiple languages, reminders, portable access, scanning/importing, flexible organisation and secure information sharing	Families reported 69 unique tasks across 9 locations using 22 storage artifacts. Frequent tasks occurred mainly in shared spaces; less frequent or time-consuming tasks occurred in private spaces.
